# Therapy of PsO in Special Subsets of Patients

**DOI:** 10.3390/biomedicines10112879

**Published:** 2022-11-10

**Authors:** Antonella Di Cesare, Federica Ricceri, Elia Rosi, Maria Thais Fastame, Francesca Prignano

**Affiliations:** Department of Health Sciences, Section of Dermatology, University of Florence, 50125 Florence, Italy

**Keywords:** psoriasis, therapy, elderly, fertility, lactation, pregnancy

## Abstract

Psoriasis is a chronic, inflammatory skin disease that may occur at any age, with a bimodal peak of incidence around the age of 16–20 years of age (early onset) and 57–60 years (late-onset). It is estimated that roughly 70% of patients develop the disease before the age of 40, which coincides with the reproductive years. Moreover, psoriasis is a chronic disease, meaning that, with increased life-duration expectancy, the number of patients affected with psoriasis aged over 65 years is going to increase and represent a big therapeutic challenge. Actually, no specific drug recommendation is available, based only on the age of the patients, while therapeutic prescription should take into account that elderly patients have more comorbidities than younger patients, with polypharmacy and an increased risk of drug interactions. Women with psoriasis are more likely to report a worse influence of the disease on their quality of life, and they are more susceptible to the development of depression. Furthermore, pregnancy and lactation represent a major contraindication to several systemic agents, and only a few studies exist providing the safety of certain drugs during these periods of life of a woman, such as certolizumab pegol. In this paper, we discuss systemic therapeutic strategies, including conventional and biological therapies, in a special subset of patients affected with moderate-to-severe psoriasis focusing on elderly patients and on female patients in fertile age, pregnancy, and lactation.

## 1. Introduction

Psoriasis is an inflammatory, immune-mediated skin disease, with a high prevalence in the general population of 2–4%, with no difference between men and women [1,2], with a bimodal peak of incidence around the age of 16–20 years (early onset) and of 57–60 years (late-onset). Roughly 70% of cases develop the disease before the age of 40, which corresponds to reproductive years [3]. 

Due to the chronic-recurrent nature of the disease, psoriasis affects a long period of the entire life-span of a single individual, with an increasing number of elderly patients (≥65 years of age) affected with psoriasis also due to the continuing rise in life expectancy of the general population [4]. Given these considerations, the therapeutic approach to psoriatic patients of different ages is challenging. Elderly patients have more comorbidities than younger patients and are more frequently treated with multiple drugs, with an increased risk of drug interactions [5].

Due to the well-known teratogenic effect of some traditional drugs for psoriasis and due to safety concerns during breastfeeding, females of fertile age, with the desire of pregnancy, or during pregnancy/lactation are frequently undertreated, both for the reluctancy of the mother that tends to consider first child’s wellbeing than her personal health and for lack of controlled studies and real-life evidence on the safety of approved systemic therapies [6].

Although pregnancy has an unpredictable effect on psoriasis, limited evidence suggests that psoriasis usually improves; around 55% improve during pregnancy, 25% report no change, and 25% worsen. Conversely, in the post-partum period, psoriasis is more likely to flare; around 65% worsen, 25% demonstrate no change, and 10% improve. It has been reported that a higher frequency of adverse outcomes, such as low birth weight, preterm delivery, pre-eclampsia, and a smaller chance for gestational age and fetal loss, are significantly associated with psoriasis in patients affected with moderate-to-severe psoriasis and/or psoriatic arthritis. This is possibly due to increased skin and serum levels of proinflammatory cytokines that are correlated to the severity of the disease [7,8,9,10,11].

Dermatologist should be trained in the management of females of fertile age to improve pregnancy outcomes and quality of life.

The aim of this paper is to discuss the current knowledge on conventional and biologic therapies in the treatment of elderly and female patients in fertile age, pregnancy, and lactation, affected with moderate-to-severe psoriasis in order to help clinicians in the clinical and therapeutic approach of a special subset of psoriatic patients.

Articles derived from a search in PubMed (https://pubmed.ncbi.nlm.nih.gov/ accessed on 1 September 2022) using “psoriasis” AND “elderly”, “geriatric”, “older”, “pregnancy”, “pregnant”, “breastfeeding”, “lactation”, “fertility”, “childbearing”. Titles and abstracts of the articles were examined, and papers that were not relevant to the paper were excluded. Thereafter, data were extracted and selected by two experts in the field of dermatology.

## 2. Therapeutic Management of Elderly Patients with Moderate-To-Severe Psoriasis

### 2.1. Conventional Therapies

#### 2.1.1. Methotrexate

The use of Methotrexate in patients aged over 65 years old is discussed. In addition to real-world data, the safety and efficacy of methotrexate (MTX) have been validated in elderly patients by clinical trials. Fairris GM et al. showed that in patients aged over 50, a dose of MTX should be based on creatinine clearance and increasing age of the patients. [12,13]. These data are confirmed by another recent study showing that the mean effective therapeutic dose of MTX administered to older patients (≥70 years old) is lower than in younger patients. Doses of 10 up to 12.5 mg/weeks appeared to be safe in this population of patients (mean dose 11.7 mg/week) [14]. Elderly patients are at risk of altered renal function as well as dehydration. Therefore, since MTX is mainly eliminated by the kidney, renal function assessment is mandatory before prescribing the drug, with dose adjustment in case of altered values or contraindication to using MTX if renal clearance of less than 60 mL/minute [15,16,17]. Therefore, elderly patients taking MTX should be reminded to stay well hydrated [18]. Psoriatic patients have an increased risk of metabolic, cardiovascular (CVD), and non-alcoholic fatty liver disease (NAFLD), which increases with the patient’s age. Actually, psoriatic patients older than 55 years are 70% more likely to have NAFLD than those without psoriasis independently of common NAFLD risk factors, and elderly psoriatic patients have a 2-fold higher risk of advanced liver fibrosis than those without psoriasis [19,20]. These data suggest that monitoring at least liver enzymes, as well as performing FibroTests and fibroscans, together with a measurement of the type III serum procollagen aminopeptide, should be considered when prescribing MTX [17,18,21,22,23,24], although there is not enough scientific evidence on the risk of cumulative dosage of MTX and liver fibrosis, that conversely is associated with obesity and NAFDL [25,26]. Psoriatic patients have an increased risk of CVD; furthermore, this risk increases with the patient’s age and increases the number of co-medications used for the treatment of CV comorbidities. Current evidence supports the protective role of MTX in CVD [23,27,28,29]. Moreover, drug interactions are not common with MTX, with the exception of trimethoprim-sulfamethoxazole and high-dose acetylsalicylic acid, which can exacerbate MTX toxicity [4,30]. Finally, MTX can potentially be associated with myelosuppression that is more likely to occur in the elderly, in patients with renal impairment or folate depletion, and with overdose or drug interactions [31]. For this reason, folic acid supplementation by 24–28 h since MTX injection should always be performed, and laboratory tests of whole blood count should be performed at one week, every month for the first three months, and routinely every three months on the long-term treatment protocols. In high-risk elderly patients, it could also be considered a starter 5 mg test dose with laboratory workup [18,32,33]. A starting dose of 10 mg/week followed by an increase to a maximum of 12.5 mg/week if PASI75 is not reached at week 12, and lowering to 7.5 mg/week in the maintenance period is a safe and effective protocol in the experience of the authors.

#### 2.1.2. Cyclosporin A

Major concerns about the use of cyclosporine A in elderly patients are associated with the safety and tolerability of the drug, i.e., the increased risk of infections and malignancies and the effects on hypertension and renal function. Elderly patients have a lower renal and cardiac reserve; therefore, side effects of cyclosporine may be more pronounced with a significantly higher incidence of renal impairment when compared to patients aged below 65 years. [18,21,22,34,35]. Increased peripheral lipid levels and overall adverse events rate (1.4/patient-year) have been associated with cyclosporin A in elderly patients compared to MTX (0.12/patient-year) [6,13]. Prescription of cyclosporin A in patients aged over 65 years should be carefully considered also due to polypharmacy for multiple comorbidities considering drug-to-drug interaction both for direct drug-to-drug effect (increased or decreased drug bioavailability, e.g., antidepressant, antibiotics, antifungal drugs, corticosteroids, statins) and for cumulative renal impairment (e.g., NSAIDs, antibiotics, colchicine) [17,21,22,36]. Starting doses from 3 to 3.5 mg/kg for four weeks followed by progressive lowering to 2–2.5 mg/kg for other eight to tweleve weeks are usually effective and well tolerated in elderly patients, when appropriate patient selection is performed at baseline, in the experience of the authors and other colleagues [14].

#### 2.1.3. Acitretin

The use of acitretin in the elderly is considered safer than younger patients especially compared to females of fertile age, due to its teratogenic effect. Moreover, due to its mechanism of action that not leads to immune suppression, it is considered a first-line treatment in patients with melanoma, solid and lymphoproliferative neoplasms, and in patients with HIV infection [37,38]. However, effects on serum lipids and on increased dryness of the skin and mucosal membranes have to be considered in elderly patients that are at higher risk of cutaneous xerosis and CVD [18,27,39]. Moreover, acitretin has proven efficacy in palmoplantar and pustular psoriasis, which are the most common forms of psoriasis seen in the elderly population after plaque-type psoriasis [40,41]. Interaction with alcohol should be underlined to patients, with summative side effects with other drugs [42]. Acitretin started at the total dose of 35 mg/day for six to eight weeks and then lowered to 20 mg/day up to 10 mg/day in the maintenance period, according to clinical response and tolerability, for up six to eight months is the used treatment protocol recommended in the psoriasis guidelines as well as by the authors.

#### 2.1.4. Dimethyl Fumarate (DMF)

Fumaric acid esters, including DMF, are one of the first-line treatments for psoriasis with a highly favorable safety profile, also in the long-term treatment. [43,44]. There is no evidence of drug–drug interactions as fumaric acid esters are not metabolized by common pathways such as cytochrome P450-dependent mono-oxygenases. Therefore, DMF can be used safely in patients with co-medication [22,45,46], such as the elderly population. Gastrointestinal events and flushing are the most common adverse events, also leading to treatment discontinuation. Liver enzyme profile and whole blood count, with close monitoring of white blood cells, are the major concerns before and during DMF monitoring. [47,48,49]. A recent study on 81 elderly psoriatic patients suggested that oral DMF intake at a standardized progressive dose regimen, starting with 30 mg of DMF, gradually increasing to a maximum daily dose of 720 mg per day, based on clinical response and tolerability, may be a first-line systemic treatment option to manage elderly psoriasis, provided that the long-term safety data are also closely monitored, lymphocytopenia in particular [50].

#### 2.1.5. Phototherapy

Phototherapy is often considered a valid option in the elderly when topical therapies are not effective or patients are not compliant with treatments or multiple comorbidities that limit systemic drug prescriptions. However, differences in skin thickness and composition between older and younger people due to reductions in epidermal turnover, melanocyte number, and tanning response which may alter photo-adaptation, have to be taken into account. UVB-induced erythema may be more persistent and become more severe at 48 h. A cautious incremental of doses is advisable [51,52]. Nonetheless, so far, no specific treatment schedules have been designed for elderly patients. Martin JA et al. showed that the safety and efficacy of PUVA and NB-UVB phototherapy in the elderly are similar to those observed in non-elderly cohorts in the short-term [53].

### 2.2. Biologic Therapies and Oral Small Molecules

Biologic therapies have become the key drugs in the treatment of moderate-to-severe psoriasis in terms of short- and long-term efficacy and safety. A huge amount of data from clinical trials, post-marketing, and real word experience have been collected; however, their use has not been sufficiently investigated in elderly patients [21,22,23,54,55,56,57].

#### 2.2.1. Anti-TNFα

The efficacy and safety of anti-TNFα agents have been extensively reported in clinical trials and observational studies. They include etanercept, adalimumab, infliximab, and certolizumab pegol either as originator or biosimilar drugs, when available. Generally, a similar efficacy in elderly patients undergoing anti-TNF has been reported so far when compared with younger patients [55,56,57,58,59], but a few studies show a lower response rate in elderly patients that were treated for other diseases than psoriasis [60]. A previous retrospective study positively evaluated the long-term efficacy and safety profile of etanercept and adalimumab in the treatment of elderly patients affected by plaque-type psoriasis and psoriatic arthritis [61]. More data on the proper use of etanercept in the elderly population were reported not only in psoriasis but also in psoriatic arthritis, rheumatoid arthritis, and other rheumatologic conditions. [62,63]. Results on adalimumab were in-line with subgroup analysis from phase III trials [64,65]. Chiricozzi et al. [66] reported a rapid PASI score reduction and maintenance in the long term in 27 elderly patients under treatment with infliximab. Recently, the efficacy of anti-TNFa in the elderly has also been confirmed for biosimilars [67].

#### 2.2.2. Anti IL-12/23

The efficacy and safety of ustekinumab in elderly patients with psoriasis were reported by Japanese and Italian authors. Hayashi M et al. showed that PASI 75 was reached in 56.5% of patients at week 16 and in 60.0% at week 52, with no relevant differences between bio-naive and bio-experienced patients [68]. Results were even more prominent in the population studied by Megna M et al., with higher percentages of patients reaching PASI 75 up to 80% of patients and maintenance of the results over a two years follow-up period [69].

#### 2.2.3. Anti IL-17

The use of IL-17 blockers (i.e., secukinumab, ixekizumab, and brodalumab) from real-world practice has been reported in a series of 114 elderly patients (mean age 72.9 years). Adverse events reported by patients were not different from the younger population, despite the higher frequency of comorbidities in this population [70]. Authors also investigated drug survival, and they found that it did not differ among the three studied drugs. These data are also confirmed by Osuna et al. The authors compared drug survival in elderly and younger patients and did not find differences related to the age of the patients [58].

#### 2.2.4. Anti-IL-23

Few data on anti-IL-23 blockers are available in patients older than 65 years. Recently, a single-center retrospective study reported positive outcomes in 34 patients treated with guselkumab, risankizumab, or tildrakizumab [23,71].

#### 2.2.5. Apremilast

No specific studies on apremilast in elderly patients are available; however, during phase-III clinical trials on psoriasis and psoriatic arthritis, apremilast was studied on more than 250 patients aged over 65 years. No overall differences were observed in the safety profile of elderly subjects compared to younger adults [72,73,74,75].

## 3. Therapeutic Management of Female Patients in Fertile Age, Pregnancy, and Lactation with Moderate-To-Severe Psoriasis

### 3.1. Conventional Therapies

#### 3.1.1. Methotrexate

The use of methotrexate during pregnancy is contraindicated due to its teratogenic effects. The FDA classifies methotrexate as Category X [8,21,22,23,76] (Table 1). Methotrexate may negatively affect embryogenesis and drive the development of “fetal methotrexate syndrome” because it inhibits dihydrofolate reductase, an essential enzyme in DNA synthesis. Methotrexate administration during pregnancy has been associated with large fontanelles, craniosynostosis, ocular hypertelorism, micrognathia, heart and limb reduction abnormalities, and developmental delay [77]. A three-month drug-free interval before conception is also recommended for both men and women.

#### 3.1.2. Cyclosporin A

Cyclosporin crosses the fetoplacental barrier and may be correlated with prematurity and low birth weight in newborns. Cyclosporine also passes into breast milk at high concentrations, and there is a risk of induced neonatal immunosuppression [78]. It is recommended to use the minimum doses only for short periods because limited data are available [8,21,22,23,79].

#### 3.1.3. Acitretin

Acitretin is in the FDAs pregnancy Category X (Table 1). It should not be used to treat pregnant women with psoriasis, and it is also recommended that the treatment be stopped three years before conceiving a child [8,21,22,23,79]. Exposure to acitretin during the first trimester of pregnancy could lead to “retionoid acid embriopathy”, a rare teratogenic disorder carrying a risk of fetal malformations, including central nervous system, craniofacial, ear, thymic, cardiac, and limb anomalies.

#### 3.1.4. DMF

Animal studies showed no evidence of teratogenicity or impaired fertility with exposure to DMF at doses that caused maternal effects. Although data are limited, there is no evidence to date of an increased risk of fetal abnormalities or adverse pregnancy outcomes associated with gestational exposure to DMF during the first trimester. Additionally, the incidence of spontaneous abortion was consistent with that reported in the general population [80,81] (Table 1).

#### 3.1.5. Phototherapy

In patients with long-term use of narrowband UVB phototherapy, a reduction in serum folate levels has been reported. Moreover, psoriatic patients may have folate deficiency, regardless of their treatment [82]. During pregnancy, maternal folate deficiency has been associated with pre-eclampsia, spontaneous abortion, stillbirth, preterm delivery, low birth weight in infants, and neural tube defects in newborns [83]. Supplementation with folic acid during pregnancy is mandatory in women patients [84].

### 3.2. Biologic Therapies and Small Oral Molecules (Table 1)

Because of their high efficacy, biologics play a central role in the treatment of patients with moderate-to-severe psoriasis, but few studies have addressed biologics treatment for psoriasis in pregnancy [8,17,21,22,23].

#### 3.2.1. Anti TNFα

Infliximab, adalimumab, and etanercept are in the FDAs pregnancy Category B [8,76,79] (Table 1). Most anti-TNFα agents (infliximab, adalimumab, golimumab, etanercept) have a classical IgG structure with an Fc region that can attach to the neonatal Fc receptor for IgG (FcRn) and cross the placenta. In particular, circulating maternal IgG antibodies are actively transported across the placenta in the second half of pregnancy, facilitated by FcRn. Importantly, IgG levels at term in the newborn are higher than maternal levels, with a half-life of approximately twice as long as that in the mother [85,86]. The Organization of Teratology and Information Specialists project has found no correlation between the administration of these drugs and the increased risk of malformations. However, during administration, monoclonal antibody levels were similar to or even higher in umbilical cord blood than in maternal serum at term [87]. A higher risk of spontaneous abortion was associated with anti-TNF-α drug treatment at the time of conception in one study, but the disease severity and the use of other drugs may also contribute to this event [88]. Concerning etanercept therapy in pregnancy, caution should be exercised as a previous case reported infants born with VATER (V: vertebral abnormalities; A: anal abnormalities; T: tracheal problems; E: esophageal problems; R: radius or renal defects) from psoriasis and psoriatic arthritis female treated with this drug during pregnancy. Because of these high concentrations in fetal blood, treatment with TNFα-inhibitors could lead to fetal and then neonatal immunosuppression. There have been several reports of fatal infections following vaccines in the first months of life (e.g., Bacillus Calmette–Guerin) [89,90]. Therefore, it has been recommended to stop biological treatments at 30 weeks of pregnancy [91]. Among anti-TNFα, certolizumab pegol differs in its specific structure allowing a different approach to fertility, pregnancy, and lactation. Certolizumab is an Fc-free pegylated anti-TNF-α drug with the lowest placental transfer rate. This structure consists of a humanized antigen-binding fragment (Fab’) of a monoclonal antibody conjugated to polyethylene glycol [92]. A prospective study on women ≥30 weeks pregnant treated with certolizumab pegol for chronic inflammatory diseases found that there was no to minimal placental transfer of certolizumab pegol from mothers to newborns [93]. It has also been found that there is a minimal plasma transfer of this drug from lactating women to breast milk [94]. Moreover, a recent study did not show an increased risk of major congenital malformations or fetal death in a group of pregnant women with chronic inflammatory diseases who received certolizumab pegol compared to the general population [95]. Although there is a need for further studies, certolizumab pegol appears to be a safe therapy for pregnancy and for the post-partum period.

#### 3.2.2. Anti IL-12/23

Ustekinumab is a monoclonal antibody against the p40 subunit of IL-12 IL-23 interleukins. It is classified by the FDA as Category B [8,76] (Table 1). Ustekinumab is a high molecular weight IgG1 molecule, and placental crossing is poor at the beginning of pregnancy and during the first weeks of pregnancy when organogenesis occurs [96], reducing the hypothetical teratogenic risks. Data on the safety of ustekinumab during pregnancy are scarce and contradictory. There are case reports of the delivery of healthy infants [97,98,99,100,101,102,103]. Ustekinumab use during breastfeeding has been approved because of the very low concentration of this drug in human milk [104].

#### 3.2.3. Anti IL-17

Interleukin 17 (IL-17) inhibitors include ixekizumab and secukinumab targeting IL-17A and brodalumab targeting IL-17RA [74]. For ixekizumab and secukinumab, data on their use during pregnancy are limited. Although animal studies have shown no adverse effects on the fetus, their use is not recommended [105,106]. In a report by Warren et al. on 238 mothers exposed to secukinumab during pregnancy, 65% discontinued it in the first trimester, and three continued it throughout their pregnancies. In these cases, no complications of pregnancy or fetal abnormalities attributable to the medication were reported [107]. Regarding brodalumab, to date, there are no studies on its safety in psoriasis pregnant or lactating; therefore, the risks and benefits must be evaluated [108].

#### 3.2.4. Anti IL-23

Biologic agents directed towards IL-23 (guselkumab, risankizumab, and tildrakizumab) represent new options for the treatment of psoriasis [109]. To date, there are no certain data on the safety of these biological drugs in the treatment of pregnant women. Risankizumab is a humanized immunoglobulin IgG1 monoclonal antibody specifically targeting the p19 subunit of interleukin IL-23 (IL-23A) [110]. Data on its use in pregnant psoriatic women are limited; therefore, it is recommended not to use this agent during pregnancy. It is also recommended to use a contraceptive method during therapy and for a minimum of 21 weeks after its suspension [111] (Table 1). No data on pregnant patients could be retrieved from clinical trials for risankizumab in psoriasis (IMMvent, UltIMMa-1 and -2, IMMhance). The long-term safety and efficacy of risankizumab in women affected by Crohn’s disease were evaluated in a phase 2 open-label extension study. In this group were reported three pregnancies, two without any complications or abnormalities and one with fetal defects [112]. Regarding Guselkumab and tildrakizumab, their use could be avoided during pregnancy; it is also recommended a contraceptive method for at least 12 weeks (guselkumab)/17 weeks (tildrakizumab) after the discontinuation of the treatment [113]. Data from pregnancies during guselkumab clinical trials showed no premature births; two spontaneous abortions were reported, involving women enrolled as healthy controls. Seven patients receiving guselkumab for psoriasis had pregnancies resulting in full-term births [114]. A post hoc analysis of data from tildrakizumab clinical trials for psoriasis revealed a total of 14 pregnancies among 528 female patients treated. In all cases, treatment was discontinued after confirmation of pregnancy. Four pregnancies resulted in elective abortion. Seven pregnancies resulted in full-term births; newborns had no anomalies except one with transient jaundice. One premature birth (36 weeks) occurred without anomalies in the newborn. Spontaneous abortions occurred in two cases, at four and eight weeks, respectively. The rate of spontaneous abortions (14%) was similar to that of the general population (12–15%). Of note, the patients whose pregnancies resulted in premature birth or spontaneous abortions did not receive any dose of tildrakizumab during pregnancy. Three patients whose pregnancies resulted in full-term births were at least 35 years old [115].

#### 3.2.5. Apremilast

There are limited data about the use of apremilast during pregnancy. Previous studies on animals did not show an increase in malformations but have shown dose-related fetal loss and reduced birth weight with the use of the drug. Apremilast was detected in the milk of lactating mice at levels approximately 1.5-fold that of blood plasma samples. It is unknown whether apremilast or its metabolites are excreted in breast milk in humans. Therefore, apremilast is contraindicated during pregnancy, and it should not be used whilst breastfeeding (Table 1). Women of childbearing potential should use effective contraception to prevent pregnancy and continue this until at least four weeks after cessation of apremilast treatment. No data are available regarding the influence of apremilast on fertility in humans [116,117].

## 4. Psoriasis and Lactation

Breastfeeding is not contraindicated in psoriatic patients, but there are some anti-psoriatic drugs that may be avoided. Psoriasis is a condition in which breastfeeding is not contraindicated, but there are some drugs used in the treatment of this disease that are contraindicated. Acitretin is excreted in small amounts in breast milk but, due to its potential for cumulative toxicity, should be avoided during breastfeeding [118,119]. The quantity of methotrexate transferred to breast milk is a small proportion of the maternal dose. However, it is reported that methotrexate accumulates in tissues, and due to the renal immaturity of the infant, its excretion may be affected. Therefore, it is contraindicated during breastfeeding [120]. The amounts of cyclosporine transferred to breast milk vary from case to case but tend to be reduced. However, it is recommended to avoid the use of cyclosporine during breastfeeding [121]. Regarding the use of biological agents, certolizumab pegol is excreted in small amounts in breast milk and is considered to be digested in the infant’s gastrointestinal tract. Experts recommend the use of certolizumab pegol during breastfeeding, and, in addition, some consider it to be a first-line therapy for moderate to severe psoriasis in lactating women [122]. Information on the use of other biologic agents during breastfeeding is limited.

## 5. Conclusions

Elderly patients, especially those with multiple comorbidities, as well as pregnant and breastfeeding females, are often excluded by selection criteria of clinical trials, with limited data available on the safety of systemic therapies in the treatment of moderate-to-severe psoriasis in these subpopulations. Current guidelines delineate a good profile for each systemic treatment; however, there is limited space to dissect all possible clinical scenarios. Focusing on special subsets of patients, as we did in this paper, should be encouraged in order to merge data from clinical trials, guidelines, and real-world experience to provide a comprehensive view of the knowledge on treatment opportunities in selected patients.

## Figures and Tables

**Table 1:** Systemic treatment options for moderate-to-severe psoriasis during pregnancy.

Drug	FDA Pregnancy Risk Categories *	Observations
**Cyclosporine**	C	**Preferred Conventional**
Metotrexate	X	Contraindicated (3 months drug-free interval before conception)
Acitretin	X	Contraindicated (3 years drug-free interval before conception)
Dimethyl-fumarate	C	Contraindicated
Apremilast	C	Contraindicated
**Anti-TNF-alpha**	B	**Certolizumab Pegol: preferred biologic**
Anti IL-12/23	B	Stopping in the second and third trimesters
Anti IL-17	B (Secukinumab) N/A (Ixekizumab; Brodalumab)	Stopping in the second and third trimesters Limited available data on use in pregnant women are insufficient to inform a drug-associated risk of adverse developmental outcomes
Anti IL-23	N/A	Limited available data on use in pregnant women are insufficient to inform a drug-associated risk of adverse developmental outcomes

FDA: Food and Drug Administration; TNF: Tumour Necrosis Factor; IL: Interleukin; N/A: Not Available; * FDA Category of Drug Evidence: A: Adequate, well-controlled studies in pregnant women do not show any risk to the fetus in the first trimester; B: Animal studies did not demonstrate risk to the fetus; no well-controlled studies in humans exist; or animal studies demonstrated risk, but well-controlled studies in pregnant women do not demonstrate adverse effects on the fetus; C: Animal studies demonstrate risk to the fetus (this category also applies to drugs for which no animal or well-controlled studies in humans exist); D: There is evidence of risk to the fetus with these drugs, but their benefits may outweigh risks; X: There is positive evidence of risk that outweighs any possible benefit.

## References

[1] World Health Organization (2016). Global Report on Psoriasis.

[2] Balato N., Patruno C., Napolitano M., Patrì A., Ayala F., Scarpa R. (2014). Managing moderate-to-severe psoriasis in the elderly. Drugs Aging.

[3] Bangsgaard N., Rørbye C., Skov L. (2015). Treating psoriasis during pregnancy: Safety and efficacy of treatments. Am. J. Clin. Dermatol..

[4] Grozdev I.S., Van Voorhees A.S., Gottlieb A.B., Hsu S., Lebwohl M.G., Bebo B.F., Korman N.J. (2011). Psoriasis in the elderly: From the medical board of the national psoriasis foundation. J. Am. Acad. Dermatol..

[5] Boots A.M., Maier A.B., Stinissen P., Masson P., Lories R.J., De Keyser F. (2013). The influence of ageing on the development and management of rheumatoidarthritis. Nat. Rev. Rheumatol..

[6] De Simone C., Calabrese L., Balato A., Cannavò S.P., Dattola A., Esposito M., Fargnoli M.C., Giuffrida R., Hansel K., Musumeci M.L. (2020). Psoriasis in Women of Childbearing Age Task Force. Psoriasis and its management in women of childbearing age: Tools to increase awareness in dermatologists and patients. G. Ital. Dermatol. Venereol..

[7] Polachek A., Li S., Polachek I.S., Chandran V., Gladman D. (2017). Psoriatic arthritis disease activity during pregnancy and the first-year postpartum. Semin. Arthritis Rheum..

[8] De Simone C., Caldarola G., Moretta G., Piscitelli L., Ricceri F., Prignano F. (2019). Moderate-to-severe psoriasis and pregnancy: Impact on fertility, pregnancy outcome and treatment perspectives. G. Ital. Dermatol. Venereol..

[9] Cohen-Barak E., Nachum Z., Rozenman D., Ziv M. (2011). Pregnancy outcomes in women with moderate-to-severe psoriasis. J. Eur. Acad. Dermatol. Venereol..

[10] Yang Y.W., Chen C.S., Chen Y.H., Lin H.C. (2011). Psoriasis and pregnancy outcomes: A nationwide population-based study. J. Am. Acad. Dermatol..

[11] Bröms G., Haerskjold A., Granath F., Kieler H., Pedersen L., Berglind I.A. (2018). Effect of Maternal Psoriasis on Pregnancy and Birth Outcomes: A Population-based Cohort Study from Denmark and Sweden. Acta Derm. Venereol..

[12] Kostović K., Žužul K., Čeović R., Bukvić Mokos Z. (2018). Psoriasis in the mature patient: Therapeutic approach in the era of biologics. Clin. Dermatol..

[13] Fairris G.M., Dewhurst A.G., White J.E., Campbell M.J. (1989). Methotrexate dosage in patients aged over 50 with psoriasis. BMJ.

[14] Piaserico S., Conti A., Lo Console F., Simone C., Prestinari F., Mazzotta A., Gualdi G., Guarneri C., Borsari S., Cassano N. (2014). Efficacy and Safety of Systemic Treatments for Psoriasis in Elderly Patients. Acta Derm. Venereol..

[15] Flammiger A., Maibach H. (2006). Dermatological drug dosage in the elderly. Ski. Ther. Lett..

[16] Kratzsch D., Treudler R. (2014). Dermatologic therapy in geriatric patients. J. Dtsch. Dermatol. Ges..

[17] Gisondi P., Fargnoli M.C., Amerio P., Argenziano G., Bardazzi F., Bianchi L., Chiricozzi A., Conti A., Corazza M., Costanzo A. (2022). Italian adaptation of EuroGuiDerm guideline on the systemic treatment of chronic plaque psoriasis. Ital. J. Dermatol. Venerol..

[18] Butler D.C., Koo J.Y.M., Chang A.L.S. (2015). Psoriasis Therapy in the Geriatric Population. Advances in Geriatric Dermatology.

[19] Van der Voort E.A., Koehler E.M., Dowlatshahi E.A., Hofman A., Stricker B.H., Janssen H.L., Schouten J.N., Nijsten T. (2014). Psoriasis is independently associated with nonalcoholic fatty liver disease in patients 55 years old or older: Results from a population-based study. J. Am. Acad. Dermatol..

[20] van der Voort E.A., Koehler E.M., Nijsten T., Stricker B.H., Hofman A., Janssen H.L., Schouten J.N., Wakkee M. (2016). Increased Prevalence of Advanced Liver Fibrosis in Patients with Psoriasis: A Cross-sectional Analysis from the Rotterdam Study. Acta Derm. Venereol..

[21] Nast A., Smith C., Spuls P.I., Avila Valle G., Bata-Csörgö Z., Boonen H., De Jong E., Garcia-Doval I., Gisondi P., Kaur-Knudsen D. (2020). EuroGuiDerm Guideline on the systemic treatment of Psoriasis vulgaris—Part 1: Treatment and monitoring recommendations. J. Eur. Acad. Dermatol. Venereol..

[22] Nast A., Smith C., Spuls P.I., Avila Valle G., Bata-Csörgö Z., Boonen H., De Jong E., Garcia-Doval I., Gisondi P., Kaur-Knudsen D. (2021). EuroGuiDerm Guideline on the systemic treatment of Psoriasis vulgaris—Part 2: Specific clinical and comorbid situations. J. Eur. Acad. Dermatol. Venereol..

[23] Lambert J.L.W., Segaert S., Ghislain P.D., Hillary T., Nikkels A., Willaert F., Lambert J., Speeckaert R. (2020). Practical recommendations for systemic treatment in psoriasis according to age, pregnancy, metabolic syndrome, mental health, psoriasis subtype and treatment history (BETA-PSO: Belgian Evidence-based Treatment Advice in Psoriasis, part 1). J. Eur. Acad. Dermatol. Venereol..

[24] Montaudié H., Sbidian E., Paul C., Maza A., Gallini A., Aractingi S., Aubin F., Bachelez H., Cribier B., Joly P. (2011). Methotrexate in psoriasis: A systematic review of treatment modalities, incidence, risk factors and monitoring of liver toxicity. J. Eur. Acad. Dermatol. Venereol..

[25] Maybury C.M., Jabbar-Lopez Z.K., Wong T., Dhillon A.P., Barker J.N., Smith C.H. (2014). Methotrexate and liver fibrosis in people with psoriasis: A systematic review of observational studies. Br. J. Dermatol..

[26] Kaushik S.B., Lebwohl M.G. (2019). Psoriasis: Which therapy for which patient: Psoriasis comorbidities and preferred systemic agents. J. Am. Acad. Dermatol..

[27] Hugh J., Van Voorhees A.S., Nijhawan R.I., Bagel J., Lebwhol M., Blauvelt A., Weinberg J. (2014). From the Medical Board of the National Psoriasis Foundation: The risk of cardiovascular disease in individuals with psoriasis and the potential impact of current therapies. J. Am. Acad. Dermatol..

[28] Mikhaylov D., Hashim P.W., Nektalova T., Goldenberg G. (2019). Systemic Psoriasis Therapies and Comorbid Disease in Patients with Psoriasis: A Review of Potential Risks and Benefits. J. Clin. Aesthet. Dermatol..

[29] Roubille C., Richer V., Starnino T., McCourt C., McFarlane A., Fleming P., Siu S., Kraft J., Lynde C., Pope J. (2015). The effects of tumour necrosis factor inhibitors, methotrexate, non-steroidal anti-inflammatory drugs and corticosteroids on cardiovascular events in rheumatoid arthritis, psoriasis and psoriatic arthritis: A systematic review and meta-analysis. Ann. Rheum. Dis..

[30] Bourré-Tessier J., Haraoui B. (2010). Methotrexate drug interactions in the treatment of rheumatoid arthritis: A systematic review. J. Rheumatol..

[31] Boffa M.J., Chalmers R.J. (1996). Methotrexate for psoriasis. Clin. Exp. Dermatol..

[32] van Huizen A.M., Menting S.P., Gyulai R., Iversen L., van der Kraaij G.E., Middelkamp-Hup M.A., Warren R.B., Spuls P.I., SPINMTXConsensus Survey Study Group, Schejtman A.A. (2022). International eDelphi Study to Reach Consensus on the Methotrexate Dosing Regimen in Patients with Psoriasis. JAMA Dermatol..

[33] Menting S.P., Dekker P.M., Limpens J., Hooft L., Spuls P.I. (2016). Methotrexate Dosing Regimen for Plaque-type Psoriasis: A Systematic Review of the Use of Test-dose, Start-dose, Dosing Scheme, Dose Adjustments, Maximum Dose and Folic Acid Supplementation. Acta Derm. Venereol..

[34] Potts G.A., Hurley M.Y. (2013). Psoriasis in the geriatric population. Clin. Geriatr. Med..

[35] Ohtsuki M., Nakagawa H., Sugai J., Ozawa A., Ohkido M., Nakayama J., Hanada J., Morimoto Y., Jimbow K., Horikoshi T. (2003). Long-term continuous versus intermittent cyclosporin: Therapy for psoriasis. J. Dermatol..

[36] Saurat J.H., Guérin A., Yu A.P., Latremouille-Viau D., Wu E.Q., Gupta S.R., Bao Y., Mulani P.M. (2010). High prevalence of potential drug-drug interactions for psoriasis patients prescribed methotrexate or cyclosporine for psoriasis: Associated clinical and economic outcomes in real-world practice. Dermatology.

[37] Ruiz-Villaverde R., Garrido-Colmenero C., Martinez-Peinado C.M., Galán-Gutierrez M., Sánchez-Cano D. (2015). Psoriasis in the elderly. Do we know how to manage it?. Hong Kong J. Dermatol. Venereol..

[38] Bhutani T., Koo J. (2011). A review of the chemopreventative effects of oral retinoids for internal neoplasms. J. Drugs Dermatol..

[39] Wong J.W., Koo J.Y.M. (2012). The Safety of Systemic Treatments That Can Be Used for Geriatric Psoriasis Patients: A Review. Dermatol. Res. Pract..

[40] Asili P., Tootoonchi N., Nasimi M., Daneshpajooh M., Sedaghatzadeh M., Mirahmad M. (2022). Demographic aspects, clinical characteristics, and therapeutic approaches in geriatric psoriasis: A study from a tertiary center. Dermatol. Ther..

[41] Lu J., Wang Y., Li Y., Gong Y., Ding Y., Shi Y. (2022). Comparative Study on the Clinical Efficacy and Safety of Acitretin and MTX in the Treatment of Pustular Psoriasis by TLR7/MyD88/CXCL16 Pathway. Appl. Bionics Biomech..

[42] Heath M.S., Sahni D.R., Curry Z.A., Feldman S.R. (2018). Pharmacokinetics of tazarotene and acitretin in psoriasis. Expert Opin. Drug. Metab. Toxicol..

[43] Mrowietz U., Altmeyer P., Bieber T., Röcken M., Schopf R.E., Sterry W. (2007). Treatment of psoriasis with fumaric acid esters (Fumaderm). J. Dtsch. Dermatol. Ges..

[44] Reich K., Thaci D., Mrowietz U., Kamps A., Neureither M., Luger T. (2009). Efficacy and safety of fumaric acid esters in the long-term treatment of psoriasis--a retrospective study (FUTURE). J. Dtsch. Dermatol. Ges..

[45] European Medicines Agency (2017). Skilarence, INN-dimethyl Fumarate: Summary of Product Characteristics.

[46] Thaci D., Weisenseel P., Philipp S., Rosenbach T., Rotterdam S., Augustin M., Neureither M., Reich K. (2013). Efficacy and safety of fumaric acid esters in patients with psoriasis on medication for comorbid conditions—A retrospective evaluation (FACTS). J. Dtsch. Dermatol. Ges..

[47] Hoefnagel J.J., Thio H.B., Willemze R., Bavinck J.B. (2003). Long-term safety aspects of systemic therapy with fumaric acid esters in severe psoriasis. Br. J. Dermatol..

[48] Wain E.M., Darling M.I., Pleass R.D., Barker J.N.W.N., Smith C.H. (2010). Treatment of severe, recalcitrant, chronic plaque psoriasis with fumaric acid esters: A prospective study. Br. J. Dermatol..

[49] Lijnen R., Otters E., Balak D., Thio B. (2015). Long-term safety and effectiveness of high-dose dimethylfumarate in the treatment of moderate to severe psoriasis: A prospective single- blinded follow-up study. J. Dermatol. Treat..

[50] Ricceri F., Bardazzi F., Buggiani G., Burlando M., Campione E., Corazza M., Cuccia A., Dapavo P., Filippi F., Zichichi L. (2022). Efficacy and safety of dimethylfumarate in elderly psoriasis patients: A multicentric Italian study. J. Dermatolog. Treat..

[51] Di Lernia V., Goldust M. (2018). An overview of the efficacy and safety of systemic treatments for psoriasis in the elderly. Expert Opin. Biol. Ther..

[52] Powell J.B., Gach J.E. (2015). Phototherapy in the elderly. Clin. Exp. Dermatol..

[53] Martin J.A., Laube S., Edwards C., Gambles B., Anstey A.V. (2007). Rate of acute adverse events for narrow-band UVB and Psoralen-UVA phototherapy. Photodermatol. Photoimmunol. Photomed..

[54] Momose M., Asahina A., Hayashi M., Yanaba K., Umezawa Y., Nakagawa H. (2017). Biologic treatments for elderly patients with psoriasis. J. Dermatol..

[55] Trettel A., Spehr C., Korber A., Augustin M. (2017). The impact of age on psoriasis health care in Germany. J. Eur. Acad. Dermatol. Venereol..

[56] Ter Haar E.L.M., Thomas S.E., van den Reek J.M.P.A., Otero M.E., Njoo M.D., Ossenkoppele P.M., Kop E.N., Dodemont S.R.P., Körver J.E.M., Kuijpers A.L.A. (2022). Drug Survival, Safety, and Effectiveness of Biologics in Older Patients with Psoriasis: A Comparison with Younger Patients-A BioCAPTURE Registry Study. Drugs Aging.

[57] Ricceri F., Bardazzi F., Chiricozzi A., Dapavo P., Ferrara F., Mugheddu C., Romanelli M., Rongioletti F., Prignano F. (2019). Elderly psoriatic patients under biological therapies: An Italian experience. J. Eur. Acad. Dermatol. Venereol..

[58] Osuna C.G., García S.R., Martín J.C., Jiménez V.G., López F.V., Santos-Juanes J. (2021). Use of Biological Treatments in Elderly Patients with Skin Psoriasis in the Real World. Life.

[59] Busquets N., Carmona L., Surís X. (2011). Systematic review: Safety and efficacy of anti-TNF in elderly patients. Reumatol. Clin..

[60] Radovits B.J., Kievit W., Fransen J., Laar M.A.F.J.V.D., Jansen T.L., Van Riel P.L.C.M., Laan R.F.J.M. (2009). Influence of age on the outcome of antitumour necrosis factor alpha therapy in rheumatoid arthritis. Ann. Rheum. Dis..

[61] Esposito M., Giunta A., Mazzotta A., Zangrilli A., Babino G., Bavetta M., Perricone R., Chimenti S., Chimenti M.S. (2012). Efficacy and safety of subcutaneous Anti-Tumor Necrosis Factor-Alpha Agents, Etanercept and Adalimumab, in elderly patients affected by Psoriasis and Psoriatic Arthritis: An observational long-term study. Dermatology.

[62] Militello G., Xia A., Stevens S.R., Van Voorhees A.S. (2006). Etanercept for the treatment of psoriasis in the elderly. J. Am. Acad. Dermatol..

[63] Fleischmann R., Baumgartner S.W., Weisman M.H., Liu T., White B., Peloso P. (2006). Long term safety of etanercept in elderly subjects with rheumatic diseases. Ann. Rheum. Dis..

[64] Poulin Y., Crowley J.J., Langley R.G., Unnebrink K., Goldblum O., Valdecantos W. (2014). Efficacy of adalimumab across subgroups of patients with moderate-to-severe chronic plaque psoriasis of the hands and/or feet: Post hoc analysis of REACH. J. Eur. Acad. Dermatol. Venereol..

[65] Menter A., Gordon K.B., Leonardi C.L., Gu Y., Goldblum O.M. (2010). Efficacy and safety of adalimumab across subgroups of patients with moderate to severe psoriasis. J. Am. Acad. Dermatol..

[66] Chiricozzi A., Pavlidis A., Dattola A., Bianchi L., Chimenti M.S., Fida M., Saraceno R. (2016). Efficacy and safety of Infliximab in psoriatic patients over the age of 65. Expert Opin. Drug. Saf..

[67] Megna M., Fornaro L., Potestio L., Luciano M.A., Nocerino M., Delfino M., Guarino M., Fabbrocini G., Camela E. (2022). Efficacy and Safety of Anti-TNF Biosimilars for Psoriasis in Pediatric and Geriatric Populations: A 72-Week Real-Life Study. Psoriasis.

[68] Hayashi M., Umezawa Y., Fukuchio O., Ito T., Saeki H., Nakagawa H. (2014). Efficacy and safety of ustekinumab treatment in elderly patients with psoriasis. J. Dermatol..

[69] Megna M., Napolitano M., Balato N., Monfrecola G., Villani A., Ayala F., Balato A. (2016). Efficacy and safety of ustekinumab in a group of 22 elderly patients with psoriasis over a 2-year period. Clin. Exp. Dermatol..

[70] Phan C., Beneton N., Delaunay J., Reguiai Z., Boulard C., Fougerousse A.C., Cinotti E., Romanelli M., Mery-Bossard L., Thomas-Beaulieu D. (2020). Effectiveness and Safety of Anti-interleukin-17 Therapies in Elderly Patients with Psoriasis. Acta Derm. Venereol..

[71] Ruggiero A., Fabbrocini G., Cinelli E., Ocampo Garza S.S., Camela E., Megna M. (2022). Anti-interleukin-23 for psoriasis in elderly patients: Guselkumab, risankizumab and tildrakizumab in real-world practice. Clin. Exp. Dermatol..

[72] Di Caprio R., Caiazzo G., Cacciapuoti S., Fabbrocini G., Scala E., Balato A. (2020). Safety concerns with current treatments for psoriasis in the elderly. Expert Opin. Drug. Saf..

[73] Crowley J., Thaci D., Joly P., Peris K., Papp K.A., Goncalves J., Day R.M., Chen R., Shah K., Ferrándiz C. (2017). Long-term safety and tolerability of apremilast in patients with psoriasis: Pooled safety analysis for >/=156 weeks from 2 phase 3, randomized, controlled trials (ESTEEM 1 and 2). J. Am. Acad. Dermatol..

[74] Papp K., Reich K., Leonardi C.L., Kircik L., Chimenti S., Langley R.G., Hu C., Stevens R.M., Day R.M., Gordon K.B. (2015). Apremilast, an oral phosphodiesterase 4 (PDE4) inhibitor, in patients with moderate to severe plaque psoriasis: Results of a phase III, randomized, controlled trial (Efficacy and Safety Trial Evaluating the Effects of Apremilast in Psoriasis [ESTEEM] 1). J. Am. Acad. Dermatol..

[75] Paul C., Cather J., Gooderham M., Poulin Y., Mrowietz U., Ferrandiz C., Crowley J., Hu C., Stevens R.M., Shah K. (2015). Efficacy and safety of apremilast, an oral phosphodiesterase 4 inhibitor, in patients with moderate-to-severe plaque psoriasis over 52 weeks: A phase III, randomized controlled trial (ESTEEM 2). Br. J. Dermatol..

[76] Pomeranz M.K., Strober B.E., Post T.W. (2022). Management of Psoriasis in Pregnancy.

[77] Nguyen C., Duhl A.J., Escallon C.S., Blakemore K.J. (2002). Multiple anomalies in a fetus exposed to low-dose methotrexate in the first trimester. Obstet. Gynecol..

[78] Ryan C., Amor K.T., Menter A. (2010). The use of cyclosporine in dermatology: Part II. J. Am. Acad. Dermatol..

[79] Paziana K., del Monaco M., Cardonick E., Moritz M., Keller M., Smith B., Coscia L., Armenti V. (2013). Ciclosporin use during pregnancy. Drug. Saf..

[80] Gold R., Phillips J.T., Havrdova E., Bar-Or A., Kappos L., Kim N., Thullen T., Valencia P., Oliva L., Novas M. (2015). Delayed-Release Dimethyl Fumarate and Pregnancy: Preclinical Studies and Pregnancy Outcomes from Clinical Trials and Postmarketing Experience. Neurol. Ther..

[81] Andersen J.B., Sellebjerg F., Magyari M. (2022). Pregnancy outcomes after early fetal exposure to injectable first-line treatments, dimethyl fumarate or natalizumab in Danish women with multiple sclerosis. Eur. J. Neurol..

[82] Shirzadian Kebria A., Hosseini M., Khafri S. (2021). The effect of narrowband ultraviolet B phototherapy on serum folate level. Casp. J. Intern. Med..

[83] Molloy A.M., Kirke P.N., Brody L.C., Scott J.M., Mills J.L. (2008). Effects of folate and vitamin B12 deficiencies during pregnancy on fetal, infant, and child development. Food Nutr. Bull..

[84] Bae Y.-S.C., van Voorhees A.S., Hsu S., Korman N.J., Lebwohl M.G., Young M., Bebo B., Kimball A.B., Foundation N.P. (2012). Review of treatment options for psoriasis in pregnant or lactating women: From the Medical Board of the National Psoriasis Foundation. J. Am. Acad. Dermatol..

[85] Mahadevan U., Wolf D.C., Dubinsky M., Cortot A., Lee S.D., Siegel C.A., Ullman T., Glover S., Valentine J.F., Rubin D.T. (2013). Placental transfer of anti-tumor necrosis factor agents in pregnant patients with inflammatory bowel disease. Clin. Gastroenterol. Hepatol..

[86] Wilmer E., Chai S., Kroumpouzos G. (2016). Drug safety: Pregnancy rating classifications and controversies. Clin. Dermatol..

[87] Gottlieb A.B., Ryan C., Murase J.E. (2019). Clinical considerations for the management of psoriasis in women. Int. J. Women’s Dermatol..

[88] Verstappen S.M., King Y., Watson K.D., Symmons D.P., Hyrich K.L. (2011). BSRBR Control Centre Consortium, BSR Biologics Register. Anti-TNF therapies and pregnancy: Outcome of 130 pregnancies in the British Society for Rheumatology Biologics Register. Ann. Rheum. Dis..

[89] Ostensen M., Förger F. (2011). Treatment with biologics of pregnant patients with rheumatic diseases. Curr. Opin. Rheumatol..

[90] Cheent K., Nolan J., Shariq S., Kiho L., Pal A., Arnold J. (2010). Case report: Fatal case of disseminated B.C.G. infection in an infant born to a mother taking infliximab for Crohn’s disease. J. Crohns Colitis..

[91] Puig L., Barco D., Alomar A. (2010). Treatment of psoriasis with anti-TNF drugs during pregnancy: Case report and review of the literature. Dermatology.

[92] Porter C., Armstrong-Fisher S., Kopotsha T., Smith B., Baker T., Kevorkian L., Nesbitt A. (2016). Certolizumab pegol does not bind the neonatal Fc receptor (FcRn): Consequences for FcRn-mediated in vitro transcytosis and ex vivo human placental transfer. J. Reprod. Immunol..

[93] Mariette X., Förger F., Abraham B., Flynn A.D., Moltó A., Flipo R.-M., van Tubergen A., Shaughnessy L., Simpson J., Teil M. (2018). Lack of placental transfer of certolizumab pegol during pregnancy: Results from CRIB, a prospective, postmarketing, pharmacokinetic study. Ann. Rheum. Dis..

[94] Clowse M.E., Förger F., Hwang C., Thorp J., Dolhain R.J., van Tubergen A., Shaughnessy L., Simpson J., Teil M., Toublanc N. (2017). Minimal to no transfer of certolizumab pegol into breast milk: Results from CRADLE, a prospective, postmarketing, multicentre, pharmacokinetic study. Ann. Rheum. Dis..

[95] Clowse M.E.B., Scheuerle A.E., Chambers C., Afzali A., Kimball A.B., Cush J.J., Cooney M., Shaughnessy L., Vanderkelen M., Förger F. (2018). Pregnancy Outcomes After Exposure to Certolizumab Pegol: Updated Results from a Pharmacovigilance Safety Database. Arthritis Rheumatol..

[96] Martin P.L., Sachs C., Imai N., Tsusaki H., Oneda S., Jiao Q., Treacy G. (2010). Development in the cynomolgus macaque following administration of Ustekinumab, a human anti-IL-12/23p40 monoclonal antibody, during pregnancy and lactation. Birth Defects Res. B Dev. Reprod. Toxicol..

[97] Andrulonis R., Ferris L.K. (2012). Treatment of severe psoriasis with ustekinumab during pregnancy. J. Drugs Dermatol..

[98] Sheeran C., Nicolopoulos J. (2014). Pregnancy outcomes of two patients exposed to ustekinumab in the first trimester. Australas. J. Dermatol..

[99] Rocha K., Piccinin M.C., Kalache L.F., Reichert-Faria A., Silva de Castro C.C. (2015). Pregnancy during Ustekinumab Treatment for Severe Psoriasis. Dermatology.

[100] Alsenaid A., Prinz J.C. (2016). Inadvertent pregnancy during ustekinumab therapy in a patient with plaque psoriasis and impetigo herpetiformis. J. Eur. Acad. Dermatol. Venereol..

[101] Galli-Novak E., Mook S.-C., Büning J., Schmidt E., Zillikens D., Thaci D., Ludwig R.J. (2016). Successful pregnancy outcome under prolonged ustekinumab treatment in a patient with Crohn’s disease and paradoxical psoriasis. J. Eur. Acad. Dermatol. Venereol..

[102] Fotiadou C., Lazaridou E., Sotiriou E., Ioannides D. (2012). Spontaneous abortion during ustekinumab therapy. J. Dermatol. Case Rep..

[103] Venturin C., Nancey S., Danion P., Uzzan M., Chauvenet M., Bergoin C., Roblin X., Flourié B., Boschetti G. (2017). Fetal death in utero and miscarriage in a patient with Crohn’s disease under therapy with ustekinumab: Case-report and review of the literature. BMC Gastroenterol..

[104] Matro R., Martin C.F., Wolf D., Shah S.A., Mahadevan U. (2018). Exposure Concentrations of Infants Breastfed by Women Receiving Biologic Therapies for Inflammatory Bowel Diseases and Effects of Breastfeeding on Infections and Development. Gastroenterology.

[105] European Medicines Agency Taltz: Summary of Product Characteristics. https://www.ema.europa.eu/en/documents/product-information/taltz-epar-product-information_en.pdf.

[106] European Medicines Agency Cosentyx: Summary of Product Characteristics. https://www.ema.europa.eu/en/documents/product-information/cosentyx-epar-product-information_en.pdf.

[107] Warren R.B., Reich K., Langley R.G., Strober B., Gladman D., Deodhar A., Bachhuber T., Bao W., Altemeyer E., Hussain S. (2018). Secukinumab in pregnancy: Outcomes in psoriasis, psoriatic arthritis and ankylosing spondylitis from the global safety database. Br. J. Dermatol..

[108] Golbari N.M., Basehore B.M., Zito P.M. (2021). Brodalumab. StatPearls.

[109] Puig L. (2017). The role of IL 23 in the treatment of psoriasis. Expert Rev. Clin. Immunol..

[110] Haugh I.M., Preston A.K., Kivelevitch D.N., Menter A.M. (2018). Risankizumab: An anti-IL-23 antibody for the treatment of psoriasis. Drug Des. Devel. Ther..

[111] European Medicines Agency Skyrizi: Summary of Product Characteristics. https://www.ema.europa.eu/en/documents/product-information/skyrizi-epar-product-information_en.pdf.

[112] Ferrante M., Feagan B.G., Panés J., Baert F., Louis E., Dewit O., Kaser A., Duan W.R., Pang Y., Lee W.-J. (2021). Long-Term Safety and Efficacy of Risankizumab Treatment in Patients with Crohn’s Disease: Results from the Phase 2 Open-Label Extension Study. J. Crohns Colitis.

[113] Owczarek W., Walecka I., Lesiak A., Czajkowski R., Reich A., Zerda I., Narbutt J. (2020). The use of biological drugs in psoriasis patients prior to pregnancy, during pregnancy and lactation: A review of current clinical guidelines. Postep. Dermatol. Alergol..

[114] Kimball A., Ferris L., Armstrong A., Song M., Ramachandran P., Lin C.B., Li S., Langley R. (2020). 15036 Pregnancy outcomes in women exposed to guselkumab: Experience from the clinical development program. J. Am. Acad. Dermatol..

[115] Haycraft K., DiRuggiero D., Rozzo S.J., Mendelsohn A.M., Bhutani T. (2020). Outcomes of pregnancies from the tildrakizumab phase I-III clinical development programme. Br. J. Dermatol..

[116] Rademaker M., Agnew K., Andrews M., Armour K., Baker C., Foley P., Frew J., Gebauer K., Gupta M., Kennedy D. (2018). The Australasian Psoriasis Collaboration. Psoriasis in those planning a family, pregnant or breast-feeding. Australas J. Dermatol..

[117] Gerosa M., Argolini L.M., Artusi C., Chighizola C.B. (2018). The use of biologics and small molecules in pregnant patients with rheumatic diseases. Expert Rev. Clin. Pharmacol..

[118] Butler D.C., Heller M.M., Murase J.E. (2014). Safety of dermatologic medications in pregnancy and lactation: Part II. Lactation. J. Am. Acad. Dermatol..

[119] Rollman O., Pihl-Lundin I. (1990). Acitretin excretion into human breast milk. Acta Derm. Venereol..

[120] Johns D.G., Rutherford L.D., Leighton P.C., Vogel C.L. (1972). Secretion of methotrexate into human milk. Am. J. Obstet. Gynecol..

[121] Belinchón I., Velasco M., Ara-Martín M., Armesto S., Rodríguez O.B., Pulido L.F., García-Bustínduy M., Martínez-López J.A., Sánchez N.M., Ferriols A.P. (2021). Management of Psoriasis During Preconception, Pregnancy, Postpartum, and Breastfeeding: A Consensus Statement. Consenso sobre las actuaciones a seguir durante la edad fértil, el embarazo, el posparto y la lactancia en pacientes con psoriasis. Actas Dermosifiliogr..

[122] (2006). Drugs and Lactation Database (LactMed).

